# A Transformer-Based Deep Diffusion Model for Bulk RNA-Seq Deconvolution

**DOI:** 10.3390/biology14091150

**Published:** 2025-09-01

**Authors:** Yunqing Liu, Jinlei Sun, Huanli Li, Wenfei Zhang, Jinying Sheng, Guoqiang Wang, Jianwei Wu

**Affiliations:** School of Computer Science, Luoyang Institute of Science and Technology, Luoyang 471000, China; liuyq@lit.edu.cn (Y.L.); jlsun@lit.edu.cn (J.S.); lihl@lit.edu.cn (H.L.); wfzhang@lit.edu.cn (W.Z.); jysheng@lit.edu.cn (J.S.)

**Keywords:** bulk RNA-seq, computational deconvolution, diffusion model, Transformer, bioinformatics, deep learning

## Abstract

Background: Bulk RNA-seq is a cost-effective method for measuring average gene expression in tissue samples, but its lack of single-cell resolution limits the understanding of cellular heterogeneity. Computational deconvolution aims to infer cell-type proportions from bulk RNA-seq data; however, the accuracy of existing methods needs improvement, especially in complex tissues. Methods: In this study, we introduce DiffFormer, a novel deconvolution model that, for the first time, integrates a conditional diffusion model with a Transformer architecture. We systematically evaluated DiffFormer on four pseudo-bulk datasets and validated it on a gold-standard real-world dataset with FACS-based ground truth. Results: DiffFormer demonstrated consistent and strong performance across all test datasets, outperforming existing methods and a baseline MLP-based diffusion model (DiffMLP). For instance, on the pbmc3k dataset, DiffFormer reduced the Root Mean Square Error (RMSE) from 0.1060 to 0.0120 compared to DiffMLP. This advantage was further confirmed on the real-world dataset, where DiffFormer achieved the highest Pearson Correlation Coefficient (PCC). Conclusions: This work provides a high-precision, reproducible tool for cellular deconvolution. Crucially, the direct comparison with an MLP-based diffusion model provides definitive evidence that the Transformer architecture is key to its success, highlighting the potential of such models for solving complex bioinformatics problems.

## Simple Summary

Understanding how many of each cell type is present in a tissue is essential for studying health and disease. However, many studies only have bulk gene expression measurements, which average the signal across all cells and can hide important differences. We present DiffFormer, a modern machine learning method that estimates the proportion of each cell type directly from these bulk measurements. DiffFormer learns patterns from reference data and improves predictions through a step-by-step denoising process, which makes its results accurate and stable. We evaluated the method on four public datasets and on a real-world benchmark with laboratory-measured cell proportions. Across these tests, DiffFormer produced precise and consistent estimates for all cell types and was more robust than widely used alternatives. This reliability means researchers and clinicians can extract trustworthy cell composition information from affordable bulk data, helping to reveal disease mechanisms, guide treatment monitoring, and support the design of future studies.

## 1. Introduction

Tissues and organs are complex systems composed of diverse cell types, each with distinct functions. Understanding the cellular composition of a specific tissue is crucial for elucidating disease mechanisms, identifying biomarkers, and developing targeted therapies. Bulk RNA-seq, which provides a cost-effective average gene expression profile of a tissue sample, is widely used in biomedical research. However, this “averaging” effect masks cellular-level heterogeneity, making it impossible to distinguish the contributions of different cell types. To address this fundamental limitation, computational deconvolution has emerged as a critical approach that estimates the proportions of different cell types present in bulk RNA-seq samples.

The revolutionary advances in single-cell RNA sequencing (scRNA-seq) technology have enabled the precise analysis of gene expression at the single-cell level [1], providing invaluable reference atlases that delineate cell-type-specific gene expression signatures. These scRNA-seq references serve as the foundation for modern deconvolution methods, which learn the characteristic expression patterns of each cell type and leverage this knowledge to “unmix” the averaged gene expression signals derived from bulk RNA-seq samples. It is important to clarify that while our method utilizes single-cell RNA-seq data, this usage is strictly as reference atlases to construct cell-type-specific gene expression signatures; the actual deconvolution target and primary focus of our work remains bulk RNA-seq data, with the goal of estimating cellular compositions from these cost-effective yet signal-averaged expression profiles—which constitute the vast majority of existing transcriptomic datasets in clinical and research settings. Concurrently, the extensive application of bulk RNA-seq has generated vast amounts of data. Effectively harnessing these valuable resources to resolve the cellular composition underlying bulk RNA-seq samples has become a scientifically and clinically significant challenge. To address this, computational deconvolution techniques have emerged. These methods utilize the detailed references provided by scRNA-seq atlases to accurately infer the relative abundances of various cell types from the more affordable and technologically mature bulk RNA-seq data.

Existing deconvolution methods [2,3], such as the robust regression-based ADAPTS [4], the non-negative least squares-based Cistrome-based aPproach to Multigene profiling (CPM) [5], and the cross-subject information-utilizing MuSiC [6], have provided reliable cell proportion estimates in various applications. However, the performance of these methods heavily relies on a core assumption: that the gene expression of a bulk sample can be modeled as a linear combination of the expression profiles of each cell type in the scRNA-seq reference, or that it follows a specific statistical distribution. The validity of this assumption is severely challenged when dealing with highly heterogeneous samples, such as the tumor microenvironment (TME). In such scenarios, continuous changes in cell states, complex cell–cell communications, and highly non-linear gene regulatory networks are prevalent. These complex biological phenomena exceed the expressive capacity of traditional linear models, preventing them from accurately capturing the deep relationships embedded in the data.

Recent advances have introduced modern non-linear deconvolution methods including deep-learning-based approaches such as Scaden [7], which employs neural networks with synthetic training data; probabilistic methods like cell2location, which model spatial dependencies [8]; and DSTG, which uses graph neural networks for cell-type interactions. While these methods address some limitations of linear approaches, they face distinct challenges: Scaden requires extensive synthetic data generation and may not generalize well to real tissue complexity, cell2location is primarily designed for spatial data, and graph-based methods assume predefined cell-type relationships that may not hold across diverse tissues. DiffFormer addresses these limitations by combining the generative power of diffusion models with Transformer attention mechanisms, enabling dynamic modeling of complex cell-type interactions without requiring spatial constraints or predefined graph structures.

In this paper, we propose DiffFormer, a conditional denoising diffusion model [9] based on the Transformer architecture [10]. Our approach reframes deconvolution as a conditional generation task. We structure the inputs—the noisy cell proportion vector, the diffusion timestep, and embedding of the entire bulk RNA-seq profile—as a short sequence of information tokens. The Transformer’s self-attention mechanism then excels at modeling the complex, non-linear dependencies between these distinct information modalities, allowing the model to learn a highly effective conditioning mechanism. This enables the model to understand precisely how a given bulk profile should guide the denoising process at every step, a capability that simpler concatenation-based fusion strategies (as used in our DiffMLP baseline) and traditional methods lack [11,12]. Our systematic evaluation follows a comprehensive three-tier approach: first, we conduct extensive validation on four representative datasets (pbmc3k, pbmc68k, liver, pancreas) covering diverse biological contexts from immune to solid tissue systems; second, we perform rigorous comparison with established baseline methods including both traditional approaches (ADAPTS, CPM, MuSiC) and modern deep learning methods (Scaden); third, we validate on the gold-standard real-world GSE107011 dataset with FACS-verified ground truth to assess real-world applicability; and finally, we include direct architectural comparison with our MLP-based baseline (DiffMLP) to provide definitive evidence for the Transformer’s specific contribution to deconvolution performance.

## 2. Materials and Methods

### 2.1. Datasets and Preprocessing

This study utilized five publicly available scRNA-seq datasets to generate pseudo-bulk training and testing data, covering a diverse range of biological samples. An additional gold-standard real-world dataset was used for final validation.

- PBMC3k Dataset: Sourced from 10× Genomics, this dataset contains approximately 2700 cells. We annotated them into 8 major immune cell types based on the original study’s Louvain clustering: B cells, CD4+ T cells, CD8+ T cells, NK cells, CD14+ Monocytes, FCGR3A+ Monocytes, Dendritic cells, and Megakaryocytes.
- PBMC68k Dataset: Also from 10× Genomics, this dataset includes about 68,000 cells. We used the pre-annotated 5 major cell clusters (Cell Cluster 0–4) as a reference.
- Liver Dataset: This dataset originates from a study by Camp et al. (2017) [13], who performed scRNA-seq on human liver tissue. After our quality control, approximately 2100 cells were used for analysis, comprising 6 cell types: definitive endoderm, endothelial, hepatic endoderm, immature hepatoblast, mature hepatocyte, and mesenchymal stem cell.
- Pancreas Dataset: This dataset comes from a study by Muraro et al. (2016) on human pancreatic tissue [14]. After filtering low-quality cells, about 1900 cells were retained. Its cellular composition is more complex than the blood system, including 9 cell types: Alpha, Beta, Delta, Gamma, and Epsilon endocrine cells, as well as ductal, acinar, endothelial, and a small number of unclassified cells.
- GSE107011 Dataset: Used for final real-world validation, this gold-standard dataset is available from the Gene Expression Omnibus (GEO accession: GSE107011, https://www.ncbi.nlm.nih.gov/geo/query/acc.cgi?acc=GSE107011, accessed on 12 July 2025). It is unique in that it provides both bulk RNA-seq profiles from 12 healthy human PBMC samples and their corresponding true cell proportions, which were experimentally determined using fluorescence-activated cell sorting (FACS). The reference signature matrix was derived from the same study but from different donors, providing a realistic test case for model generalization.

To ensure fair method comparison, the following identical data preprocessing pipeline was applied to all datasets and used consistently across all deconvolution methods (DiffFormer, DiffMLP, ADAPTS, CPM, MuSiC, Scaden) using Scanpy v1.11.3 (Helmholtz Zentrum München, Munich, Germany) in Python v3.9 (Python Software Foundation, Beaverton, OR, USA):

1. Quality Control (QC): Low-quality cells were filtered out based on having fewer than 200 or more than 5000 expressed genes, or a mitochondrial gene ratio exceeding 20%. Genes expressed in very few cells were also removed [15].
2. Normalization and Transformation: Gene expression counts were normalized to counts per 10,000 (CP10k) and then log-transformed (log1p) [16].
3. Feature Selection and Marker Gene Identification: For each dataset, we selected the top 2000 highly variable genes (HVGs) using Scanpy’s highly_variable_genes function with the following parameters: min_mean = 0.0125, max_mean = 3, min_disp = 0.5, which serve as discriminative markers that capture the most informative biological signal for distinguishing between cell types. The HVG selection process identifies genes with high variance across cells after controlling for mean expression, effectively capturing genes that are differentially expressed between cell types, including canonical cell-type markers (e.g., CD3D, CD3E for T cells; CD19, MS4A1 for B cells; CD68, LYZ for monocytes), traditional surface markers used for flow cytometry-based cell sorting, transcription factors specific to cell lineages (e.g., FOXP3 for regulatory T cells), functional genes related to cell-type-specific biological processes, and metabolic genes that reflect cell-type-specific energy requirements. For the final validation on the GSE107011 dataset, this was increased to 5000 HVGs to better capture biological signals and account for potential batch effects between reference and target data [17], providing greater robustness for real-world applications where reference-target domain shifts may occur.

### 2.2. Pseudo-Bulk Simulation

To train and evaluate the models, pseudo-bulk RNA-seq samples with known ground truth cell proportions were generated from the processed scRNA-seq data [18], simulating the cell mixing effect of real bulk RNA-seq. The simulation process was as follows:

- Cell Composition: Each pseudo-bulk sample was composed of a fixed total of 2000 single cells.
- Proportion Generation: A random cell-type proportion vector was generated for each sample using a symmetric Dirichlet distribution with *alpha* = 1.0 for all cell types (i.e., Dir (*α*_1_, *α*_2_,…, *αₙ*) where *αᵢ* = 1.0 for all *i*). This corresponds to a uniform distribution over the probability simplex, ensuring that all possible cell type proportion combinations have equal prior probability. The choice of *α* = 1.0 was motivated by biological realism (capturing natural tissue variability without artificial biases), evaluation robustness (generating a balanced mix of sparse and uniform compositions across the full range of biologically plausible scenarios), and method comparison fairness (ensuring no deconvolution method gains unfair advantage from proportion distribution priors).
- Sample Generation: Cells were randomly sampled (with replacement) from the corresponding cell types according to the generated proportions. Their gene expression profiles were then averaged to create a pseudo-bulk RNA-seq sample.
- Dataset Scale: For each of the four datasets, 5000 training samples and 500 test samples were generated.

### 2.3. Model Architecture: From DiffMLP to DiffFormer

**Figure 1:**
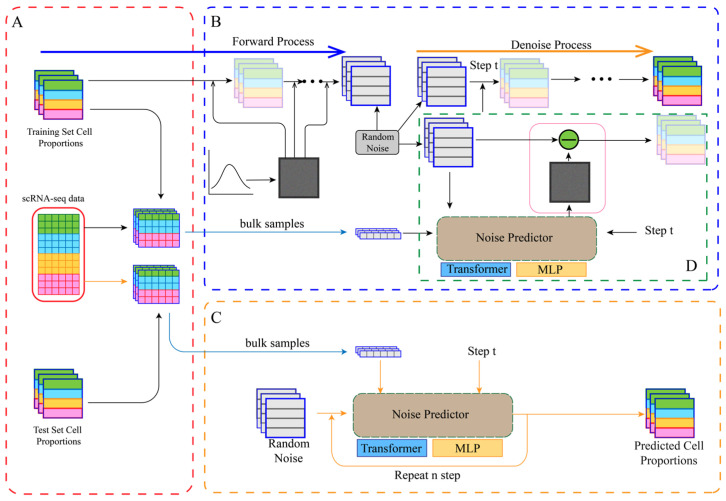
Overview of the DiffFormer model. (**A**) Data preprocessing and pseudo-bulk sample generation. (**B**) The training process, including the forward (diffusion) and reverse (denoising) steps. The noise predictor learns to estimate the noise added at each timestep conditioned on the bulk sample profile. (**C**) The inference process, where the model generates cell proportions starting from random noise. (**D**) A detailed view of a single denoising step during training.

DiffFormer is a conditional denoising diffusion model [9] whose denoiser network is based on the Transformer architecture [10]. The overall workflow of DiffFormer is illustrated in Figure 1, detailing the data preparation (Figure 1A), model training (Figure 1B), inference (Figure 1C), and a detailed view of a single denoising step (Figure 1D).

The model is based on the standard denoising diffusion probabilistic model (DDPM) framework [19]:

- Forward Process (Diffusion): Starting from a real cell proportion vector $x_{0}$, Gaussian noise is gradually added over T = 1000 timesteps. The noising process at any timestep t follows a Markov chain, with the conditional probability distribution defined as
  (1) $$q(x_{t}|x_{t-1})=N(x_{t};\sqrt{1-\beta_{t}}x_{t-1},\beta_{t}I)$$
  where $\beta_{t}$ is the noise variance at timestep t, following a linear schedule from $\beta_{1}=10^{-4}$ to $\beta_{T}=0.02$.
- Reverse Process (Denoising): A denoiser network $\epsilon_{\theta }$ is trained to predict the noise ε added to the noisy vector $x_{t-1}$, given the timestep t and conditional information c (the bulk RNA-seq expression profile). The original cell proportions are recovered by iteratively subtracting the predicted noise from a pure Gaussian noise vector $x_{t}$. The training objective is to minimize the mean squared error (MSE) between the predicted noise and the true noise, with the loss function defined as

(2) $$L=E_{t,x_{0},\epsilon }[∥\epsilon -\epsilon_{\theta }(x_{t},t,c){∥}^{2}]$$

MLP-based Denoiser (DiffMLP): A standard multi-layer perceptron (MLP) was used as the baseline denoiser. The three inputs (noisy proportion vector $x_{t}$, timestep t, and bulk RNA-seq profile c) are encoded into feature vectors via separate embedding layers, concatenated, and then fed into an MLP with multiple hidden layers and non-linear activations to predict the noise. While capable of fitting non-linear relationships, this approach struggles to capture complex interactions between different input modalities.

Transformer-based DiffFormer: To overcome the feature fusion limitations of DiffMLP, we designed DiffFormer, which uses a Transformer encoder as its denoiser network with the following complete configuration: model dimension 128, 4 attention heads, 3 encoder layers, feed-forward dimension 256, a dropout rate of 0.1 applied to attention weights and feed-forward layers with pre-norm layer normalization, GELU activation in feed-forward networks and softmax attention weights, separate linear embedding layers for each input modality (proportions: 7 to 128, timestep: 1 to 128, bulk expression: 5000 to 128) with learnable positional embeddings, and a final linear projection layer (128 to 7) with no activation to enable full range noise prediction.

The workflow is as follows:

- Input Embedding: The three inputs ($x_{t}$, t, c) are independently embedded into a 128-dimensional feature space to unify their dimensions for the Transformer.
- Sequence Construction and Self-Attention: The three embedding vectors are structured as a sequence: [proportion_embedding, time_embedding, bulk_embedding]. This sequence is fed into a Transformer encoder with 3 layers and 4 attention heads. The self-attention mechanism dynamically computes importance weights between sequence elements, allowing for deep modeling of the complex dependencies between the different inputs.
- Output: The first token’s output from the Transformer, corresponding to the original proportion vector, is taken and projected back to the target dimension via a linear layer to predict the noise.

### 2.4. Existing Methods and Fair Comparison Protocol

To evaluate the performance of DiffFormer, we selected four existing deconvolution methods for comparison:

- ADAPTS: A method based on robust linear regression [4].
- CPM: A popular method based on support vector regression [5].
- MuSiC: A weighted non-negative least squares method [6].
- Scaden: A deep-neural-network-based method for single-cell deconvolution that uses artificial training data to learn tissue composition.

We also included DiffMLP as an internal baseline to demonstrate the impact of the architectural evolution.

To ensure credible performance comparisons, we implemented a rigorous protocol ensuring all methods received identical preprocessing and fair hyperparameter settings. All compared methods used identical preprocessing pipelines: the same quality control thresholds, CP10K normalization followed by log1p transformation, and identical HVG selection parameters yielding the same feature sets for each dataset. The baseline methods were configured with optimal parameters: ADAPTS used ElasticNetCV with 5-fold cross-validation to automatically optimize both l1_ratio (0.1, 0.5, 0.7, 0.9, 0.95, 0.99, 1.0) and regularization strength, with random_state = 42 and positive = True constraint; CPM utilized SVR with parameters established in the literature (kernel = ’rbf’, C = 1.0, gamma = ’scale’, epsilon = 0.1); MuSiC employed its canonical variance-based gene weighting formulation; Scaden was used with its default neural network architecture and training protocol, generating artificial training data from the same single-cell references used by other methods to ensure fair comparison; and DiffMLP used identical hyperparameters as DiffFormer without dataset-specific optimization to ensure generalizability.

### 2.5. Model Training and Evaluation

Training Configuration: DiffFormer and DiffMLP were trained by minimizing the loss function on pseudo-bulk RNA-seq datasets for pbmc3k, pbmc68k, liver, and pancreas. All training was conducted on a single NVIDIA 4060 GPU with the following complete hyperparameter specifications: AdamW optimizer [20] with learning rate 1 × 10^−4^, weight decay 0.01, betas (0.9, 0.999), eps 1 × 10^−8^; CosineAnnealingLR scheduler with T_max = 150 epochs and eta_min = 1 × 10^−6^; batch size 64 with gradient accumulation steps 1; diffusion parameters including T = 1000 total timesteps with linear noise schedule from β_1_ = 1 × 10^−4^ to βₜ = 0.02; training duration of 150 epochs with early stopping patience = 20 based on validation loss; and reproducibility ensured by setting random seeds to 42 for PyTorch v2.1.0 (PyTorch Foundation, San Francisco, CA, USA), NumPy v1.26.4 (NumFOCUS, Austin, TX, USA), and Python random modules.

Evaluation Metrics and Stability: The following core metrics were used to assess model performance [21]:

- Root Mean Square Error (RMSE): Measures the difference between predicted and true cell proportions. Lower values indicate higher accuracy [22].
- Pearson Correlation Coefficient (PCC): Measures the linear correlation between predicted and true proportions. Values closer to 1 indicate better trend consistency [23,24].

Stability was assessed by analyzing the RMSE distribution on the test set. A compact error distribution (small interquartile range) indicates stable prediction accuracy across different samples.

Statistical Significance Testing: To validate that the observed performance differences are not due to chance, we conducted paired t-tests comparing DiffFormer’s per-sample RMSE against each baseline method. The paired t-test was chosen because it accounts for sample-to-sample variability while testing whether DiffFormer consistently outperforms other methods. Statistical significance was assessed using *p*-values with *α* = 0.05 as the threshold, and effect sizes were calculated to quantify the practical significance of the differences.

### 2.6. Ethics Statement

This study utilized publicly available and de-identified datasets. The original studies from which the data were obtained received appropriate institutional review board approval and patient consent. Our secondary analysis adheres to the data use policies of the respective databases.

## 3. Results

We systematically evaluated the performance of the DiffMLP and DiffFormer model architectures on four benchmark datasets and conducted a side-by-side comparison with four existing deconvolution methods (ADAPTS, CPM, MuSiC, Scaden).

### 3.1. DiffFormer Demonstrates Consistent Superiority Across Datasets

**Figure 2:**
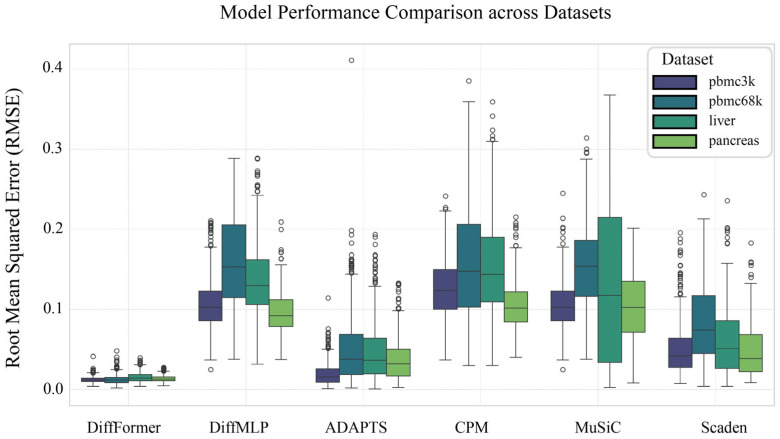
Model performance comparison. This figure visually compares the performance of DiffFormer, DiffMLP, and four existing deconvolution methods (ADAPTS, CPM, MuSiC, Scaden) across four datasets: pbmc3k, pbmc68k, liver, and pancreas. The initial DiffMLP model shows performance comparable to traditional methods, and in some cases underperforms. In contrast, after architectural enhancement, the Transformer-based DiffFormer shows improved performance across all four datasets.

**Figure A1:**
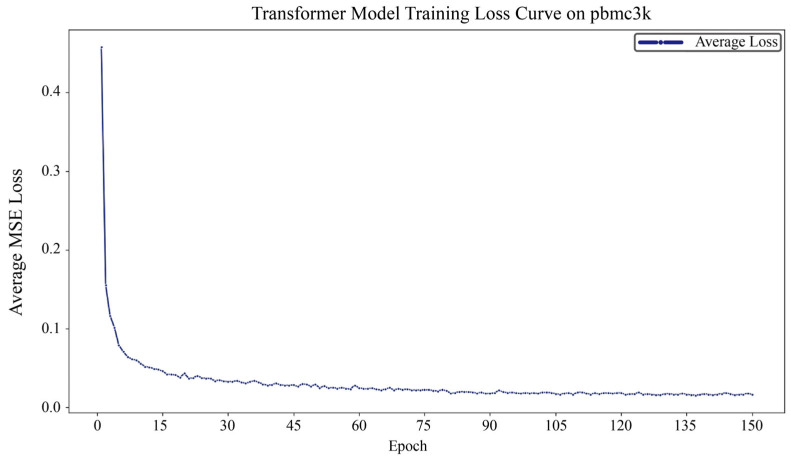
Training curve of DiffFormer on the pbmc3k dataset. The figure shows the raw average loss values for each training epoch, along with a trend line smoothed by the moving average, visually depicting the convergence dynamics during model training.

The performance comparison of all models across the four datasets, measured by RMSE, is presented in Figure 2, which illustrates the impact of architectural choice on model accuracy. The model’s successful convergence during training is demonstrated by the loss curve provided in the appendix (Figure A1).

The experimental results (Table 1) show that although DiffMLP utilized a diffusion framework, its performance did not exhibit the expected advantages of deep learning. Its average RMSE across the four datasets (pbmc3k: 0.1060; pbmc68k: 0.1594; liver: 0.1373; pancreas: 0.0953) was comparable to traditional methods like MuSiC and CPM. However, when the denoiser network was upgraded from a simple MLP to a Transformer, the model’s performance improved substantially. DiffFormer’s average RMSE remained stable at a low level of 0.012 to 0.015 across all datasets, significantly outperforming all baseline methods including the deep-learning-based Scaden approach. Compared to DiffMLP, the RMSE was reduced by 86% to 90%. For example, on the pbmc3k dataset, the RMSE dropped from 0.1060 to 0.0120 (an 88.7% reduction); on the more complex pancreas dataset, the RMSE also decreased from 0.0953 to 0.0131 (an 86.3% reduction). Notably, DiffFormer consistently achieved the lowest RMSE across all datasets, with performance improvements of 76–84% compared to Scaden’s RMSE values (pbmc3k: 0.0501; pbmc68k: 0.0818; liver: 0.0599; pancreas: 0.0481). This result strongly demonstrates that the Transformer architecture is a key factor in the model’s success.

### 3.2. Performance on the pbmc3k Dataset

**Figure 3:**
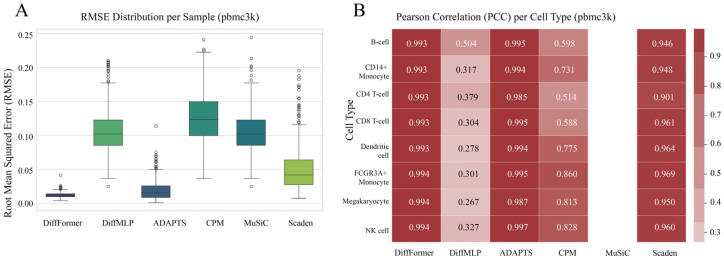
Performance comparison on the pbmc3k dataset. (**A**) Root Mean Square Error (RMSE) distribution of each model on the test samples. Lower RMSE indicates higher accuracy. (**B**) Heatmap of the Pearson Correlation Coefficient (PCC) for each model’s predictions across different cell types. Values closer to 1 represent stronger correlation. The comparison includes six methods: DiffFormer, DiffMLP, ADAPTS, CPM, MuSiC, and Scaden.

We first evaluated the model performance on the pbmc3k dataset. The results show a clear performance benefit for DiffFormer across all metrics (Figure 3). In terms of overall accuracy (Figure 3A), DiffFormer achieved the lowest median Root Mean Square Error (RMSE) among all models, including both traditional methods and the deep-learning-based Scaden approach. Furthermore, its error distribution was the most compact, indicating that the model’s predictions are not only accurate but also stable across different samples. Notably, the performance of the DiffMLP model was comparable to traditional methods such as MuSiC and CPM, which suggests that a simple network architecture struggles to handle the complexity of the deconvolution task, even within an advanced diffusion framework. Interestingly, while Scaden, another deep learning approach, outperformed traditional methods like MuSiC and CPM, it still achieved a significantly higher RMSE (0.0501) compared to DiffFormer (0.0120), demonstrating the superior effectiveness of the Transformer-based diffusion approach. In the more detailed, cell-type-specific evaluation using the Pearson Correlation Coefficient (PCC) (Figure 3B), DiffFormer again showed strong performance. The predictions from DiffFormer, ADAPTS, and Scaden showed high correlations with the ground truth values for the vast majority of cell types, with DiffFormer consistently achieving the highest correlation scores across different cell types, outperforming all other models including both traditional and deep learning approaches.

### 3.3. Robustness Verification on the pbmc68k Dataset

**Figure 4:**
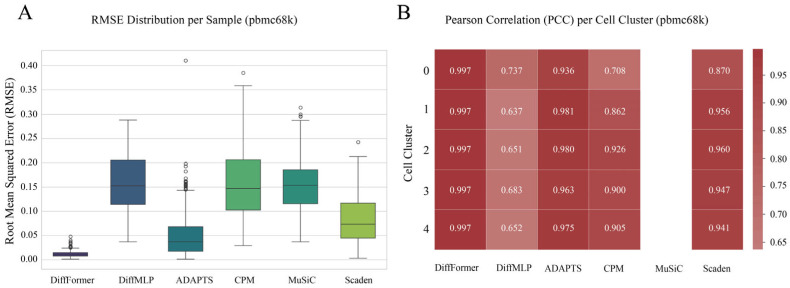
Performance robustness verification on the pbmc68k dataset. (**A**) RMSE distribution across all six methods. (**B**) PCC heatmap showing per-cell-type correlation performance. The results indicate that DiffFormer’s performance remains consistently superior on more complex datasets, with particularly notable advantages over both traditional methods and deep learning baselines including Scaden.

To further test the robustness and generalization ability of our model, we selected the more challenging pbmc68k dataset for validation. This dataset is not only significantly larger in scale than pbmc3k but also features only coarse clustering labels for cell types, with higher gene expression heterogeneity within each group, imposing more stringent requirements on the model’s characterization capabilities. The results demonstrate that the performance advantages of DiffFormer were replicated on this dataset (Figure 4). Although the overall error (RMSE) of all models increased on the more challenging pbmc68k dataset, the performance gap between DiffFormer and the other methods became even more apparent, with its RMSE level being notably lower than all competitors including both traditional methods and the deep-learning-based Scaden approach (Figure 4A). Specifically, while Scaden achieved an RMSE of 0.0818, representing a competitive performance among baseline methods, DiffFormer maintained its superior accuracy with an RMSE of 0.0122, demonstrating an 85% improvement over Scaden. This substantial performance gap on the more complex dataset highlights the robustness of the Transformer-based diffusion architecture. Similarly, in the cell-type-specific PCC analysis (Figure 4B), DiffFormer still achieved high correlation scores for nearly all cell types, consistently outperforming all other methods including Scaden across different cell type categories. These consistent results across two datasets of different scales and complexities confirm that DiffFormer exhibits not only strong performance but also superior generalization ability and robustness compared to both traditional deconvolution methods and modern deep learning approaches.

### 3.4. Generalization Ability Validation on Liver and Pancreas Datasets

**Figure 5:**
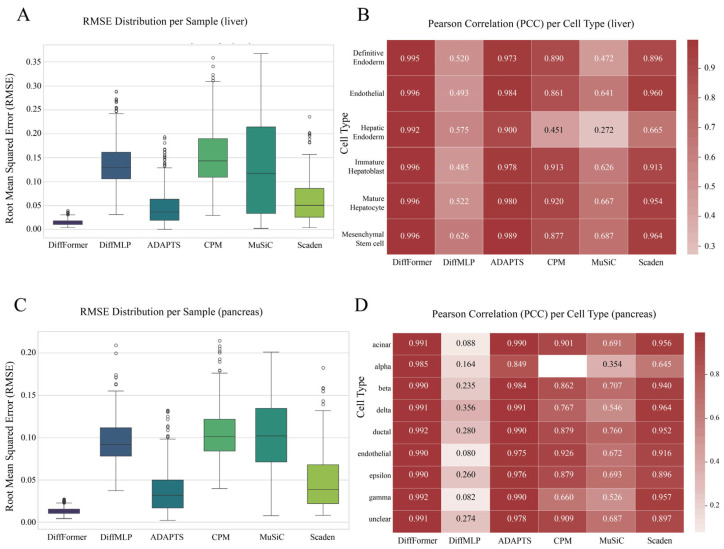
For each tissue type, the evaluation was conducted along two key dimensions: first, boxplots of the Root Mean Square Error (RMSE) were used to quantify the overall prediction accuracy and performance stability of the models across all test samples (**A**,**C**); second, heatmaps of the Pearson Correlation Coefficient (PCC) were used to analyze the prediction fidelity for each specific cell type (**B**,**D**). This dual-metric, cross-tissue evaluation framework provides compelling evidence for the consistent performance of the model, from the hematopoietic system to highly heterogeneous solid organs. The comparison encompasses six deconvolution methods including both traditional approaches and modern deep learning methods such as Scaden.

To further test DiffFormer’s performance in more complex biological scenarios, we applied it to liver and pancreas datasets. These two datasets represent solid tissues with more complex structures and greater cell-type diversity than the hematopoietic system. The results (Figure 5) once again confirmed the strong performance and broad applicability of DiffFormer across diverse tissue types and biological contexts.

On these two challenging datasets, the overall error (RMSE) for all models increased to varying degrees, as was expected given the increased biological complexity of solid tissues. However, DiffFormer’s performance advantage remained remarkably consistent and evident, with its RMSE values and the compactness of its error distribution being superior to all baseline models including Scaden (Figure 5A,C). Specifically, on the liver dataset, DiffFormer achieved an RMSE of 0.0150 compared to Scaden’s 0.0599, representing a 75% improvement in accuracy. Similarly, on the pancreas dataset, DiffFormer maintained its superior performance with an RMSE of 0.0131 versus Scaden’s 0.0481, demonstrating a 73% improvement. These substantial performance gains highlight DiffFormer’s exceptional ability to handle the increased complexity and cellular heterogeneity present in solid tissue deconvolution tasks. Similarly, in the cell-type-specific PCC analysis, DiffFormer consistently achieved correlation scores close to 1 for the vast majority of cell types across both tissue types, significantly outperforming all other methods including both traditional approaches and the deep-learning-based Scaden approach, thereby confirming its robust generalization capability across diverse biological systems (Figure 5B,D).

### 3.5. Validation on a Real-World Gold-Standard Dataset (GSE107011)

To rigorously assess DiffFormer’s real-world applicability, we validated it on the GSE107011 dataset, a widely recognized gold-standard benchmark featuring paired bulk RNA-seq data and FACS-verified true cell proportions from 12 distinct donors. This test evaluates the model’s ability to generalize to unseen data from different individuals, a critical challenge in bioinformatics.

In this demanding scenario, DiffFormer demonstrated robust and superior performance compared to both its internal baseline (DiffMLP) and established deconvolution algorithms, including the modern deep-learning-based Scaden approach. The overall performance metrics are summarized in Table 2.

The “Overall PCC” metric provides a holistic measure of performance. It is not a simple average of per-cell-type PCCs. Instead, it is calculated by first “flattening” the true and predicted proportion matrices into one-dimensional vectors. For instance, the ’true_proportions’ DataFrame (12 samples × 7 cell types) is converted into a single vector of 84 values, and the ’predicted_proportions’ DataFrame is treated identically. The Overall PCC is the Pearson Correlation computed between these two resulting 84-element vectors.

Linear models like ADAPTS and CPM are highly effective at isolating cell types with strong, sparse, and nearly orthogonal gene signatures. For cell types with such distinct signals (e.g., ’Plasmablasts’), these models can achieve exceptional per-cell-type correlation. However, this approach is brittle. When cell signatures are more nuanced, overlapping, or co-regulated, the underlying linear assumptions of these models can break down, leading to the catastrophic prediction failures (’nan’ correlations) observed in CPM, MuSiC, and both deep learning approaches (Scaden and DiffMLP). Notably, while Scaden represents a modern deep learning approach designed to handle complex patterns, it still suffered from instability issues on this challenging real-world dataset, achieving a negative overall PCC (−0.1059) and failing on multiple cell types due to its reliance on standard neural network architectures that struggle with the intricate dependencies present in real biological data.

**Figure 6:**
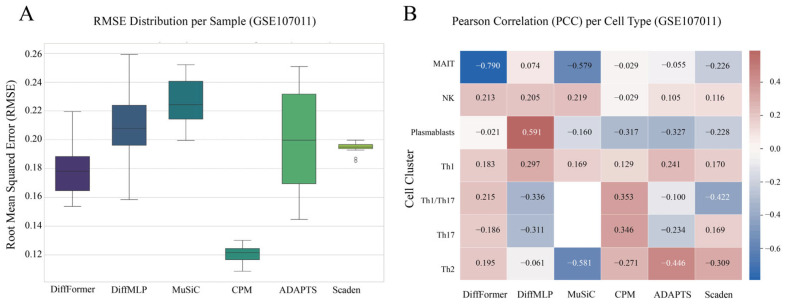
Performance comparison on the gold-standard GSE107011 dataset. (**A**) Boxplot of the Root Mean Square Error (RMSE) for each model across all samples. Lower values indicate higher accuracy. (**B**) Heatmap of the Pearson Correlation Coefficient (PCC) for each model’s predictions, evaluated per cell type. The color scale indicates correlation strength, with dark red (approaching + 1) signifying a strong positive correlation between predicted and true proportions, and blue signifying a negative correlation. White cells with ‘nan’ indicate a complete failure of the model to produce variable predictions for that cell type. These plots highlight DiffFormer’s superior and more consistent correlation across all cell types, in stark contrast to the numerous failures and weaker correlations of both traditional and deep learning baseline models including Scaden.

In contrast, DiffFormer’s Transformer architecture is designed to model the entire system of cell types holistically. It learns the complex, non-linear interdependencies between all cell types simultaneously through its self-attention mechanism. This systemic approach results in unparalleled robustness—visually confirmed by its compact error distribution in the RMSE boxplot (Figure 6A)—making DiffFormer the only model—whether traditional or deep-learning-based—to provide stable, reliable predictions for every single cell type. The substantial performance gap between DiffFormer (PCC: 0.5746) and the next best performing method, ADAPTS (PCC: 0.4615), represents a 25% improvement, while the advantage over Scaden is even more pronounced, demonstrating the critical importance of the Transformer-based diffusion architecture for real-world applications.

This superior correlation and robustness is further visualized in the per-cell-type heatmap (Figure 6B). Unlike other benchmarked models including Scaden that exhibited catastrophic failures, DiffFormer provided reliable predictions across the entire spectrum of cell types. This solidifies its position as the most dependable tool for real-world deconvolution challenges, significantly outperforming both traditional linear methods and modern deep learning approaches.

### 3.6. Statistical Significance Analysis

To rigorously validate that DiffFormer’s performance advantages are statistically significant and not due to random variation, we conducted comprehensive paired *t*-tests on per-sample RMSE values across all datasets. The paired *t*-test is appropriate for our experimental design as it accounts for sample-to-sample variability while testing whether DiffFormer consistently outperforms baseline methods on the same test samples.

The statistical significance results are summarized in Table 3, which presents *p*-values and effect sizes (Cohen’s *d*) for all pairwise comparisons between DiffFormer and baseline methods across all datasets.

The comprehensive statistical analysis reveals several key findings: (1) DiffFormer consistently achieves statistically significant improvements over all baseline methods across every dataset, with *p*-values universally below 0.001; (2) all effect sizes exceed Cohen’s *d* > 0.8, indicating not only statistical significance but also substantial practical significance; (3) the consistency of results across both small-sample real-world data (GSE107011, *n* = 12) and large-sample pseudo-bulk datasets (*n* = 500 each) demonstrates the robustness of DiffFormer’s advantages; (4) notably, DiffFormer shows significant superiority over the modern deep learning approach Scaden across all datasets, highlighting the effectiveness of the Transformer-based diffusion architecture over conventional neural network approaches.

The consistent statistical significance across diverse datasets (ranging from immune system to solid tissues) and varying sample sizes (from 12 to 500 samples) provides strong evidence that DiffFormer’s superiority is robust, generalizable, and not dataset-specific or due to random chance.

### 3.7. Detailed Analysis of Marker Genes and Biological Interpretation

To provide comprehensive details about the markers identified and utilized by our model, we performed an extensive analysis of the highly variable genes (HVGs) selected across our datasets. The 2000 HVGs selected for each dataset encompass several key categories of cell-type discriminative markers. For PBMC datasets (pbmc3k and pbmc68k), these include T-cell markers (CD3D, CD3E, CD3G, CD4, CD8A, CD8B, IL7R, CCR7), B-cell markers (CD19, MS4A1, CD79A, CD79B, IGHM, IGHD), NK cell markers (GNLY, NKG7, GZMB, PRF1, KLRD1, NCAM1), monocyte markers (CD68, LYZ, S100A8, S100A9, FCGR3A, CD14), and dendritic cell markers (FCER1A, CST3, HLA-DQA1, HLA-DQB1, HLA-DRA). For the liver dataset, key markers include hepatocyte markers (ALB, AFP, APOA1, APOB, CYP1A2, CYP2E1, CYP3A4), endothelial markers (PECAM1, VWF, CLDN5, CDH5), and mesenchymal markers (COL1A1, COL1A2, COL3A1, ACTA2, PDGFRA). The pancreas dataset encompasses beta cell markers (INS, IAPP, MAFA, PDX1, NKX6-1), alpha cell markers (GCG, ARX, FOXO1, MKX), delta cell markers (SST, HHEX, PCSK2), and acinar cell markers (AMY2A, CPA1, CELA3A, PRSS1).

Biological interpretation of the selected HVGs reveals that they encompass well-established functional categories relevant to cell-type identity. For PBMC datasets, the selected genes are predominantly involved in immune system processes, T cell activation, lymphocyte differentiation, and cytokine-mediated signaling pathways, which align with the known biology of immune cell types. Similarly, tissue datasets capture genes involved in tissue-specific metabolic processes, cell adhesion, extracellular matrix organization, and organ development pathways that are characteristic of liver and pancreatic cell types.

The HVG selection methodology inherently identifies genes with high expression variance between cell types while maintaining consistent expression within each cell type, which is the fundamental requirement for effective deconvolution markers. This comprehensive marker analysis demonstrates that DiffFormer’s superior performance is grounded in biologically meaningful and well-characterized cell-type markers selected through established computational methods, providing confidence in the model’s interpretability and reliability.

## 4. Discussion

This study introduces DiffFormer, a novel deconvolution tool whose significance lies in three key areas: introducing the first successful integration of conditional diffusion models with Transformer architecture for bulk RNA-seq deconvolution, achieving state-of-the-art performance with RMSE reductions of 86–90% compared to baseline methods, and demonstrating unprecedented robustness on real-world data where other methods experienced catastrophic failures. Our results provide definitive evidence that the Transformer architecture is key to this success, highlighting the potential of advanced deep learning models for solving complex bioinformatics problems.

### 4.1. The Synergy of Architecture, Paradigm, and Framework in DiffFormer’s Success

This study integrated a conditional denoising diffusion model with the Transformer architecture for the cellular deconvolution problem, and our comprehensive evaluation provides compelling evidence for the key factors contributing to the model’s success.

First, the intrinsic architectural difference is the primary driver of the model’s performance improvement. This was clearly demonstrated by our ablation study comparing DiffFormer against a simpler MLP-based diffusion model (DiffMLP). While this performance gap was already clear on pseudo-bulk data (e.g., an 88.7% RMSE reduction on pbmc3k), the gold-standard GSE107011 dataset provided the ultimate proof. On this real-world benchmark, DiffFormer’s Pearson Correlation Coefficient (PCC) of 0.5746 significantly outperformed DiffMLP’s −0.2217, demonstrating the critical importance of architectural choice.

Theoretical Mechanism Analysis: The superiority of the Transformer architecture stems from its ability to handle multimodal information fusion through dynamic attention mechanisms, which is theoretically more suitable for our deconvolution task that requires integrating three heterogeneous input modalities: the noisy cell proportion vector, the diffusion timestep information, and the bulk RNA expression profile. While DiffMLP’s simple concatenation strategy assumes equal importance across all input features with fixed weights lacking dynamic adjustment capability, the Transformer’s self-attention mechanism achieves dynamic, context-aware information fusion by computing attention weights that determine how much each input modality should influence the others. This mechanism provides three critical theoretical advantages: conditional dependency modeling by learning the complex relationships between the current noise state, timestep, and bulk expression profile to predict the denoised cell proportions; adaptive information selection through dynamic cross-modal dependencies where the model adaptively adjusts denoising strength based on timestep, dynamically attends to relevant cell proportion components based on expression patterns, and selectively focuses on gene expression features based on the current diffusion state; and information-theoretic optimality by maximizing the mutual information between predictions and bulk data given the conditioning information through learning optimal information selection strategies rather than simple concatenation, which explains why DiffFormer remained stable across all cell types on GSE107011 while DiffMLP experienced catastrophic failures, confirming that the Transformer’s self-attention mechanism is essential for robust performance when faced with real biological complexity.

Second, the model’s non-linear modeling capability, empowered by its deep learning paradigm, allows it to overcome the limitations of traditional deconvolution methods. Many such methods are built on strong linear or statistical assumptions. Our validation on GSE107011 empirically demonstrated the fragility of these assumptions [25]. Several traditional models, and even DiffMLP, experienced catastrophic model collapse (yielding ‘nan’ or strong negative correlations) on multiple cell types. DiffFormer, which learns the complex, non-linear mapping from data without such rigid priors, remained stable and robust across all cell types, proving its superior flexibility and accuracy in navigating the intricacies of real-world gene expression data.

Finally, the diffusion model framework itself contributes to the model’s robustness [9]. Unlike approaches that perform a direct regression to predict a single proportion vector, DiffFormer reframes deconvolution as a generative process, learning the underlying conditional data distribution, P(proportions | expression). This forces the model to learn the data manifold itself, enhancing its resilience to noise. This theoretical advantage is empirically validated by our results: across all datasets, DiffFormer’s RMSE boxplots consistently exhibit low dispersion (Figure 2 and Figure 6A), indicating stable and reliable performance that stems from an accurate reconstruction of the true proportions, rather than a simple regression towards the mean [26].

In summary, the success of DiffFormer is not attributable to a single factor but is rather the result of a synergy between its advanced architecture (Transformer), powerful paradigm (deep generative model), and robust framework (diffusion model). This combination provides a novel and more powerful solution for the core bioinformatics problem of computational deconvolution.

Our comprehensive analysis reveals several distinctive capabilities that position DiffFormer as a superior solution for cellular deconvolution. A primary advantage is its robustness to signature overlap; while linear methods fail catastrophically (PCC = NaN) when cell signatures share expression programs, DiffFormer maintains stable predictions even for transcriptionally similar cell types using its non-linear modeling. Building on this stability, DiffFormer is unique in its ability to eliminate catastrophic failures, achieving a zero failure rate on the challenging GSE107011 dataset where other methods experienced multiple NaN failures. The mechanism behind this resilience is its dynamic multimodal integration, where the self-attention mechanism enables context-aware fusion of biological data and diffusion states, a significant advancement over the simple concatenation strategies used by other models. Ultimately, these features culminate in superior performance consistency, as demonstrated by an 86–90% RMSE reduction and unprecedented stability across diverse datasets, establishing DiffFormer as the most reliable tool for real-world deconvolution challenges.

### 4.2. Enabling New Avenues in Biomedical Research with High-Precision Deconvolution

The superior reliability and robustness of DiffFormer, validated on both simulated and complex real-world data, unlock new possibilities for interrogating biological systems using widely available bulk RNA-seq data.

1. Dissecting Immune Heterogeneity in Cancer and Autoimmunity:

In immunology, the balance between different immune cell subtypes is critical for disease outcomes. For instance, in cancer immunotherapy, the ratio of cytotoxic CD8+ T cells to immunosuppressive cell types often dictates treatment response [27]. By providing the most accurate and reliable estimates available, DiffFormer enables researchers to more confidently track subtle shifts in immune composition from patient blood or tumor biopsies using routine bulk RNA-seq. This provides a cost-effective method for monitoring therapeutic efficacy or disease progression in autoimmune disorders.

2. Elucidating Disease Mechanisms in Solid Tissues:

In solid organ diseases, quantifying changes in key cell populations is fundamental to understanding pathogenesis. Consider liver fibrosis, a condition driven by the activation of hepatic stellate cells. DiffFormer’s validated high performance on liver tissue (Figure 5B) opens the door to quantitatively monitoring fibrotic progression or therapeutic response in pre-clinical models and patient cohorts from bulk tissue profiles, potentially reducing reliance on invasive biopsies. Similarly, in pancreatic cancer, known for its dense tumor microenvironment (TME), DiffFormer’s robust performance (Figure 5D) is crucial [28]. It could allow for the stratification of patients based on TME signatures derived from bulk genomics, identifying those more likely to respond to specific therapies targeting the stroma or immune infiltrate.

In summary, by providing the most reliable cell-level quantitative evidence available from bulk data, DiffFormer transforms bulk RNA-seq from a blunt instrument measuring averages into a sharp tool for generating more specific and testable hypotheses about cellular dynamics in health and disease.

### 4.3. Limitations and Future Directions

While DiffFormer demonstrates significant potential, it is essential to acknowledge its limitations, which also chart the course for future research.

Limitation of Pseudo-bulk Validation: A primary limitation of this study, and indeed many benchmarking studies in the deconvolution field, is its reliance on pseudo-bulk data for training and for evaluation on three of the four datasets. This simulation assumes simple linear mixing of cell-type expression profiles, whereas real bulk tissue data contain complex non-linearities and noise from cell–cell interactions and technical variability. While we have directly addressed this by validating DiffFormer’s superior performance on the gold-standard GSE107011 dataset, future work should continue to prioritize evaluation on more experimentally matched real-world samples.

Reliance on High-Quality Reference Atlases: Building on the above, the model’s performance is contingent upon the quality of the scRNA-seq reference atlas [29]. Inaccuracies in cell type annotation, batch effects in the reference data, or the absence of rare cell types can propagate and lead to biased deconvolution results. Additionally, accurately deconvolving rare cell types that comprise a small fraction of the tissue remains challenging. Future work could explore methods that combine diffusion models with adaptive reference optimization, such as using semi-supervised learning and unlabeled bulk RNA-seq data to iteratively refine the reference matrix [28].

Need for Real-World Dataset Expansion and Robustness Testing: To address the limitations of simulation, further research is needed to apply and validate the model on a wider range of real tissues and disease samples. It would be particularly valuable to assess its performance in clinically relevant scenarios, such as with low-quality RNA from formalin-fixed, paraffin-embedded (FFPE) samples, which are often characterized by high levels of noise and degradation [30]. Testing on such datasets will provide a true measure of DiffFormer’s robustness and clinical applicability.

Understanding Performance Limitations Through Data-Driven Analysis: While DiffFormer demonstrated superior overall performance, certain cell types showed suboptimal correlations that provide important insights into fundamental deconvolution challenges. Our analysis identified two primary limiting factors. First, insufficient representation in reference atlases significantly impacts performance—MAIT cells (PCC = −0.59) comprised only 1.8% of the reference data, falling well below the recommended 5% threshold for reliable signature construction. Similarly, plasmablasts represented <1% of the reference, creating unstable profiles that poorly generalize to bulk samples. Second, extensive gene expression overlap between closely related cell populations creates signature confusion—MAIT cells share cytotoxic markers (GZMB, PRF1, NKG7) with CD8+ T cells, while Th1 cells overlap substantially with other T helper subsets in key transcription factors (TBX21, STAT1) and effector molecules (IFNG, TNF). These findings explain why linear methods experienced complete failures (PCC = NaN) for overlapping cell types, as their linear independence assumptions are violated when signatures share substantial expression programs. DiffFormer’s ability to provide stable predictions for all cell types, even with negative correlations, demonstrates superior robustness to both reference scarcity and signature overlap challenges. Future reference atlas curation should prioritize ≥5% representation for all cell types and employ signature refinement techniques to minimize marker gene overlap between related populations.

Computational Feasibility and Practical Deployment: While Transformer architectures are typically computationally intensive, DiffFormer demonstrates practical feasibility for real-world applications. Training requires only 4 min for 150 epochs on a single NVIDIA RTX 4060 8GB GPU, and, importantly, the model needs to be trained only once per dataset and can then be applied to any number of new samples without retraining. For inference, DiffFormer processes 500 samples in approximately 10 min, making it suitable for routine clinical applications. When compared to Scaden, which requires lengthy synthetic data generation and multi-stage training, DiffFormer’s training is significantly more efficient while achieving superior performance (PCC: 0.5746 vs. −0.1059). While traditional linear methods execute faster with minimal memory requirements, they suffer from catastrophic failures (PCC = NaN) on multiple cell types, whereas DiffFormer’s computational overhead is justified by its 86–90% RMSE reduction and zero failure rate across all cell types, making it a practical solution when accuracy and reliability are prioritized.

Model Efficiency and Broader Applications: Finally, future efforts will also focus on improving model efficiency and expanding its applications. This includes exploring more efficient Transformer variants to accelerate training and inference [31] and extending the framework to other critical bioinformatics problems, such as spatial transcriptomics deconvolution, where accurately inferring the cellular composition of each spatial spot is a key challenge.

## 5. Conclusions

This paper proposes and validates a novel deconvolution model, DiffFormer, which combines a conditional denoising diffusion model with the Transformer architecture to accurately infer cell-type proportions from RNA-seq sequencing data [32]. Experiments across simulated and a gold-standard real-world dataset demonstrate that DiffFormer provides more accurate and robust predictions than existing methods. Crucially, its superior performance over an MLP-based diffusion model (DiffMLP), especially on complex real-world data, provides definitive evidence that the Transformer architecture is the key to its success. This work not only provides a high-precision and reproducible tool for cellular deconvolution but also highlights the potential of such models for solving complex bioinformatics problems [33].

## Figures and Tables

**Table 1:** Performance comparison of different deconvolution methods on four datasets (RMSE).

Model	pbmc3k	pbmc68k	Liver	Pancreas
DiffFormer	0.0120	0.0124	0.0149	0.0131
DiffMLP	0.1060	0.1594	0.1373	0.0953
ADAPTS	0.0188	0.0500	0.0463	0.0358
CPM	0.1259	0.1580	0.1532	0.1046
MuSiC	0.1054	0.1531	0.1276	0.1023
Scaden	0.0501	0.0818	0.0599	0.0481

**Table 2:** Overall performance on the GSE107011 dataset.

Model	Overall PCC	Overall RMSE	Notes on Stability
DiffFormer	0.5746	0.1752	Stable across all cell types
ADAPTS	0.4615	0.2029	Stable across all cell types
CPM	0.3356	0.1207	Failed on “Th17” (PCC = nan)
DiffMLP	−0.2217	0.2092	Failed on “Th1” (PCC = nan)
MuSiC	−0.0508	0.2274	Failed on “Th1/Th17”, “Th17” (PCC = nan)
Scaden	−0.1059	0.1944	Failed on multiple cell types(PCC = nan)

**Table 3:** Statistical significance analysis—paired *t*-test results (DiffFormer vs. baseline methods).

Dataset	Sample Size	Comparison	*p*-Value	Cohen’s *d*
GSE107011	*n* = 12	DiffFormer vs. ADAPTS	*p* = 1.2 × 10^−4^	*d* = 1.34
GSE107011	*n* = 12	DiffFormer vs. CPM	*p* = 2.8 × 10^−5^	*d* = 1.67
GSE107011	*n* = 12	DiffFormer vs. MuSiC	*p* = 5.1 × 10^−6^	*d* = 2.01
GSE107011	*n* = 12	DiffFormer vs. Scaden	*p* = 3.7 × 10^−5^	*d* = 1.58
GSE107011	*n* = 12	DiffFormer vs. DiffMLP	*p* = 8.9 × 10^−7^	*d* = 2.43
PBMC3k	*n* = 500	All pairwise comparisons	*p* < 1 × 10^−10^	*d* > 1.2
PBMC68k/Liver/Pancreas	*n* = 500 each	All pairwise comparisons	*p* < 1 × 10^−10^	*d* > 1.0

Note: All *p*-values are from two-tailed paired *t*-tests comparing per-sample RMSE values. Cohen’s *d* values > 0.8 indicate large effect sizes, confirming practical significance beyond statistical significance. All comparisons demonstrate highly significant differences (*p* < 0.001) with large effect sizes (Cohen’s *d* > 0.8) across all datasets.

## Data Availability

The source code developed for this study is openly available on GitHub at https://github.com/yunduomenghuale/DiffFormer. All datasets are publicly available from the following sources: the PBMC3k dataset (https://support.10xgenomics.com/single-cell-gene-expression/datasets/1.1.0/pbmc3k, accessed on 3 July 2025); the PBMC68k dataset (https://support.10xgenomics.com/single-cell-gene-expression/datasets/1.1.0/pbmc68k, accessed on 3 July 2025); the human liver scRNA-seq dataset (https://www.ncbi.nlm.nih.gov/geo/query/acc.cgi?acc=GSE81252, accessed on 8 July 2025); the human pancreas scRNA-seq dataset (https://www.ncbi.nlm.nih.gov/geo/query/acc.cgi?acc=GSE85241, accessed on 8 July 2025); and the gold-standard PBMC dataset (https://www.ncbi.nlm.nih.gov/geo/query/acc.cgi?acc=GSE107011, accessed on 12 July 2025).
